# The Detoxification and Degradation of Benzothiazole from the Wastewater in Microbial Electrolysis Cells

**DOI:** 10.3390/ijerph13121259

**Published:** 2016-12-20

**Authors:** Xianshu Liu, Jie Ding, Nanqi Ren, Qingyue Tong, Luyan Zhang

**Affiliations:** State Key Laboratory of Urban Water Resource and Environment, School of Municipal and Environmental Engineering, Harbin Institute of Technology, Harbin 150090, China; liu_xianshu@126.com (X.L.); dongding8493@163.com (Q.T.); zly3161@163.com (L.Z.)

**Keywords:** benzothiazole, microbial electrolysis cell, potential toxicity, biodegradability

## Abstract

In this study, the high-production-volume chemical benzothiazole (BTH) from synthetic water was fully degraded into less toxic intermediates of simple organic acids using an up-flow internal circulation microbial electrolysis reactor (UICMER) under the hydraulic retention time (HRT) of 24 h. The bioelectrochemical system was operated at 25 ± 2 °C and continuous-flow mode. The BTH loading rate varied during experiments from 20 g·m^−3^·day^−1^ to 110 g·m^−3^·day^−1^. BTH and soluble COD (Chemical Oxygen Demand) removal efficiency reached 80% to 90% under all BTH loading rates. Bioluminescence based Shewanella oneidensis strain MR-1 ecotoxicity testing demonstrated that toxicity was largely decreased compared to the BTH wastewater influent and effluent of two control experiments. The results indicated that MEC (Microbial Electrolysis Cell) was useful and reliable for improving BTH wastewater treatment efficiency, enabling the microbiological reactor to more easily respond to the requirements of higher loading rate, which is meaningful for economic and efficient operation in future scale-up.

## 1. Introduction

Benzothiazole derivatives (BTHs) are a group of xenobiotic heterocyclic chemicals that contain a benzene ring fused with a thiazole-ring, and are high-production-volume chemicals found in medical intermediates, corrosion inhibitors. Evidence for leaching of BTH and other benzothiazoles is also provided by leaching from drug preparations, rubber materials, herbicides, slimicides, algicides, fungicides, photosensitizers, azo dyes, de-icing/anti-icing fluids, and food flavors [1,2,3,4]. These applications clearly indicate that BTHs have a wide spectrum of biological activity. Released from factories producing and using BTHs, these compounds have been detected in industrial wastewaters [5] and in various environmental compartments, such as soil, groundwater, and surface water, via drainage systems [6,7] at concentrations ranging from a few ng/L to hundreds of mg/L, depending on the compound and the matrix. The limited biodegradability of BTHs, their potential toxicity toward microorganisms [8,9], their allergenicity [10], and their potential mutagenic effects [11] make their presence in the environment a great concern. These compounds have therefore already been classified as emerging organic pollutants, and some are considered ubiquitous water contaminants, which has led to the establishment of a maximum limit for TTR (Tolyltriazole, 7 ng/L) and pTTR (p-Tolyltriazole, 300 ng/L) in the drinking water guidelines of Australia [12] and Germany [13], respectively.

Acute aquatic toxicity of BTHs has been reported [4,14]; either BTH itself or a toxic intermediate product prevents its further degradation [14]. In addition, in studies focusing on their removal during wastewater and sewage treatment, it has been observed that BTHs are not effectively removed by conventional treatments. BTHs are structurally similar to naturally occurring purines, as they can interact with charged biomolecules [15]. This structural similarity of BTHs with purines may pose a risk for humans if it triggers circulation of BTH derivatives in the human body, as has been suggested for certain perfluorinated acids that are similar to fatty acids (i.e., enter hepatic circulation) [15,16].

Few researchers have reported on the biodegradation of BTH, and then only with Gram-positive strains represented by *Rhodococcus* spp. [14] reported that *Rhodococcus erythropolis* is able to degrade 2-hydroxybenzothiazole, benzothiazole-2-sulfonate, and BTH, but not 2-mercaptobenzothiazole (MBT). Biodegradation pathways of BTH, 2-hydroxybenzothiazole, and MBT have been partially elucidated with the *Rhodococcus pyridinovorans* strain PA [9] and the *Rhodococcus rhodochrous* strain OHBT [17]. The degradation of 2-aminobenzothiazole by *R. rhodochrous* was recently reported [18,19]. El-Bassi et al. [20] reported the transformation of BTH by the Gram-negative bacterium *Pseudomonas putida* strain HKT554 via the oxidization of the thiazole-ring of BTH to form benzothiazolone/2-hydroxybenzothiazole. Unfortunately, conventional biological wastewater treatment processes could not effectively remove such contaminants since they are resistant to biodegradation and tend to adsorb on cell membrane, leading to bio-accumulation [1,21].

In comparison to other conventional BTH removal strategies, microbial electrolysis system is attracting global attention for its higher degradation efficiency, lower maintenance cost, and more environmental sustainability for pollutants treatment [22,23,24]. Within the MEC (Microbial Electrolysis Cell) reactor, refractory compounds might be oxidized/reduced and then be further relieved of biotic resistance, as an oxidation and a reduction process would occur at the anode and the cathode, respectively [22,25]. Additionally, the coupling of microorganisms and current might achieve better MEC performance, which could get over the limitations of electron transfer from electrodes to microorganism, and thereby help to reduce the biological overpotentials of those stubborn compounds [26]. Moreover, taking organic wastes as a carbon source might be another option to further cut down the MEC operating costs, as the organic wastes are both abundant and easily accessible. Recently, MEC has been studied extensively for hydrogen production and the reductive degradation of various recalcitrant pollutants [27,28]. Although MEC was claimed to be capable of degrading antibiotic such as sulfonamides, ceftriaxone, and penicillin [29,30,31], no report has been published on the feasibility of using MEC technology for removing antibacterial activity and enhancing the biodegradability of BTH.

Furthermore, electrode materials and reactor design are two serious issues associated with scaling-up of MECs. To further lower the overpotential and the overall internal resistance, catalysts are always needed; platinum (Pt) is the best choice in respect of high catalysis activity and has been widely used in traditional MECs studies. However, it is well accepted that Pt is not feasible for up-scaling application due to the high cost and negative environmental impacts, and carbon-based electrodes represent an alternative source of candidates due to their good stability and low cost.

Putting it all together, an up-flow internal circulation microbial electrolysis reactor (UICMER) is developed here as a potential platform technology to detoxify and degrade of BTH, and potentially treat wastewater. It provided an up-flow pattern of MEC reactor, which improved the mass transfer efficiency by making the wastewater pass through the cathode and the anode in turn, compared to the conventional MEC reactors. Furthermore, graphite material, carbon-based electrodes with good stability and low cost, are used in this reactor, which makes it possible for application on an industrial scale.

In this study we demonstrated that the BTH removal efficiency in the MEC was significantly enhanced and the BTH reduction rate accelerated with an open circuit reactor as a control. The results presented in this article are part of a wider ongoing project on the fundamental study of the degradation of toxic organic compounds using MEC.

## 2. Materials and Methods

### 2.1. Experimental Setup

In order to study the performance of BTH degradation by micro-organisms in the presence of electricity, three reactors were designed, a reactor with external power and biomass, a reactor with only biomass, and a reactor with only external power.

**Figure 1:**
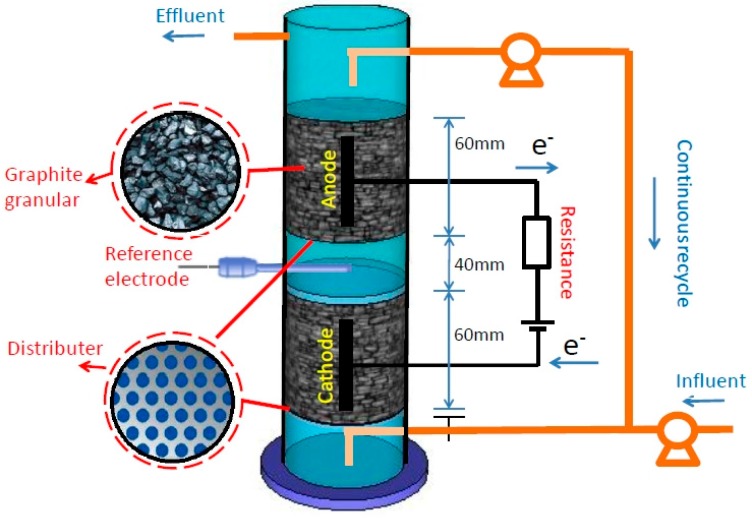
Schematic diagram of an up-flow internal circulation microbial electrolysis reactor (UICMER).

The schematic diagram of the UICMER for BTH degradation is shown in Figure 1. In brief, the reactor, which had a total liquid volume of 650 mL, was constructed from a cylindrical acrylic plastic tube chamber (φ90 mm × 250 mm), with a cathode zone at the bottom and an anode zone on the top without a membrane. Graphite granules (150 g) ranging from 3–5 mm were stacked as cathode and anode. A pair of graphite rods (φ10 mm× 60 mm) was inserted into the center of the bed of granules respectively as current collectors connected with a power supply (3645 A, Array Elec. Co., Ltd., Nanjing, China) of 0.7 V through a titanium wire (1 mm in diameter, Baoji Lixing Titanium Group Co., Ltd., Yuxing, China) (hereafter referred to as R1). The voltage across a high-precision resistor (10 Ω) in the circuit was measured using a multimeter/data acquisition system (Model 2700 with 7702 Module, Keithley, Inc., Washington, WA, USA) at 20-min intervals to calculate current. A saturated calomel reference electrode (SCE, 0.247 V vs. standard hydrogen electrode (SHE), Model-217, Shanghai Precise. Sci. Instrument Co., Ltd., Shanghai, China) was placed between the anode and the cathode to measure half potentials; here the electrode potential was reported against SCE. The solution was driven by a pump to circulate continuously between the upper anode section and the lower cathode section.

The control experiments were conducted in the following two reactors, a MEC reactor that was the same as R1, but without the external power (hereafter referred to as R2), and a MEC reactor that was the same as R1, except without the biological sludge (hereafter referred to as R3).

### 2.2. Microbial Inoculum and Wastewater

Microbial consortium used as the inoculum of the Biological electrodes was preenriched using activated sludge collected from Wastewater Treatment Plant of Harbin Pharmaceutical Group Co., Ltd. (Harbin, China). The acclimated activated sludge was fed into the reactor to immerse the ceramic plates for 24 h to form a preliminary biofilm by adsorption, and then excess sludge was discharged out. The ratio of volatile suspended sludge to total suspended sludge (VSS/TSS) of the sludge was 0.72. The sludge was added to the two reactors (R1 and R2) with an initial TSS of 11.0 g/L. An artificial BTH wastewater composed of benzothiazole (BTH in abbreviation, that is a typical widely used pharmaceutical intermediate) was employed in this study. Glucose and BTH were added to the wastewater as the source of carbon, NH_4_Cl and KH_2_PO_4_ were added to the wastewater as the nitrogen and phosphorus sources, respectively, to give a COD (Chemical Oxygen Demand):N:P ratio of 200:5:1. The trace elements were added according to the following composition: 1 mL/L of a trace element solution containing Zn at 0.37 mM/L, Mn at 2.5 mM/L, Cu at 0.14 mM/L, Co at 8.4 mM/L, Ni at 0.25 mM/L, H_3_BO_3_ at 0.8 mM/L, and EDTA (Ethylene Diamine Tetraacetic Acid) at 3.4 mM/L [32]. The pH of the influent wastewater was adjusted to 7.0 using a phosphate buffer solution (PBS; 50 mM, pH = 7) [33].

### 2.3. Operation

After being seeded (except R3), three reactors were fed with artificial wastewater with a constant COD of 2000 mg/L and BTH of 20 mg/L with a hydraulic retention time (HRT) of 24 h under a continuous mode, and a fixed voltage of 0.7 V was applied to the MECs. These three reactors were operated at about 25 °C, which was selected according to the temperature of wastewater with large amount of BTH in a pharmaceutical enterprise in Harbin. After 16 days of operation, the COD removal and BTH degradation in the three reactors were gradually stabilized. Thereafter, the reactors were fed using the following waters with a fixed COD at 2000 mg/L (include glucose and BTH) under a fixed voltage of 0.7 V through experimental course in turn: (1) wastewater with BTH concentration gradually increasing from 20 to 110 mg/L for all three reactors under a continuous mode with a hydraulic retention time (HRT) of 24 h; (2) wastewater with BTH concentration fixed at 60 mg/L for all reactors under a batch mode with a hydraulic retention time (HRT) of 48 h over multiple cycles (five times) to investigate the BTH biodegradation pathway at a fixed voltage of 0.7 V. Each time MECs was replenished with fresh medium, and samples were taken at time intervals to analyze BTH concentrations. As a control, R3 was operated in batch with inactivated biofilm, which was prepared by immersing the electrodes (granular-graphite and the graphite plate) into boiling water for 15 min to inactivate the bacteria.

### 2.4. Analysis

The analyses of total COD, SCOD, TSS, and VSS were conducted according to standard methods (APHA, 2005). The concentrations of VFAs (Volatile Fatty Acid, such as acetic, propionic, butyric, and valeric acids) and ethanol were analyzed using another gas chromatograph (7890A, Agilent, Inc., Santa Clara, CA, USA). The pH was recorded using a pH analyzer (Sartorius PB-20, Goettingen, Germany). Benzothiazole concentration was measured using a high performance liquid chromatograph (Model e2695, Waters Co., Milford, MA, USA) under ultraviolet detection set at 254 nm with a ZORBAX SB-C18 column (5 μm, 4.6 mm × 150 mm, Agilent). The mobile phase was a mixture of methanol: water solution (55:45, *v*/*v*), and the flow rate was 1 mL/min.

The extracellular polymeric substances (EPS) were extracted using a cation exchange resin. Polysaccharides in the EPS were evaluated by the sulfuric acid-anthrone method and protein in the EPS was analyzed according to the method described by Miron et al. [34].

### 2.5. Antibacterial Activity Measurement

After reaction had progressed for 24 h, most BTH in reactors was consumed. The samples (300 mL, influent and effluent) were withdrawn from the reactors and then centrifugated at 13,000 rpm for 30 min to collect supernatant. The supernatant was then dried by lyophilization. The residues were dissolved into 10 mL methanol, which was then concentrated to a final volume of 600 μL. The concentrated methanol solution was filtered via a sterile 0.22 μm filter prior to antibacterial activity tests. The antibacterial activity of BTH and its catabolism to bacterial strains was evaluated based on their toxicity on the viabilities of Shewanella oneidensis strain MR-1 (an electrogenic bacterium). Cells of the strain were harvested in their exponential growth phase, washed for three times using sterile phosphate buffer solution (PBS) (50 mM, pH 7.0), and then inoculated into 50 mL of 3-fold-diluted Luria-Bertani (LB) medium with the same cell concentrations based on optical density at 600 nm (OD600 nm) by a spectrometer (UV2550, Shimadzu, Kyoto, Japan). Both BTH and the degradation products were added to the 3-fold-diluted LB medium. The same medium without BTH served as control. The antibacterial activity assay was performed in triplicates. During the antibacterial activity assay tests, samples were collected every 3 h. Biomass was collected by centrifugation of 1 mL cell suspension samples at 13,000 rpm for 10 min and the pellets were resuspended in the 1 mL PBS (50 mM; pH 7.0) to measure OD600 nm (Shimadzu UV2550 Spectrometer, Shimadzu, Kyoto, Japan).

## 3. Results and Discussion

### 3.1. COD Removal and BTH Degradation at Different BTH Concentrations

The experiments were initially carried out to investigate the effects of the electrodes on microbial degradation of BTH and COD removal at different BTH concentrations.

**Figure 2:**
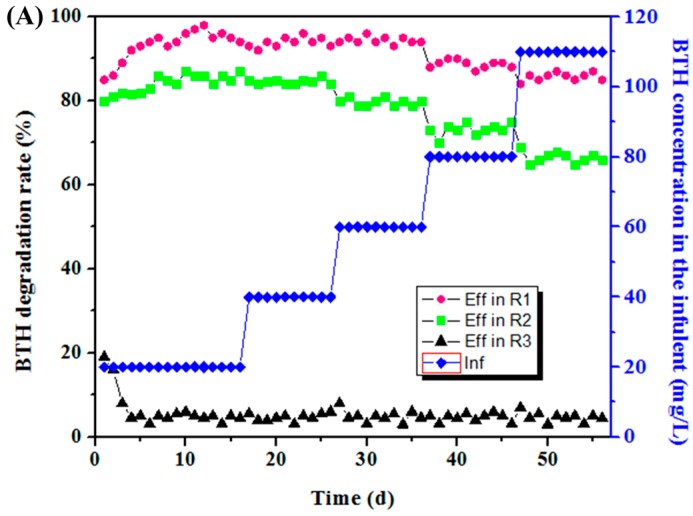

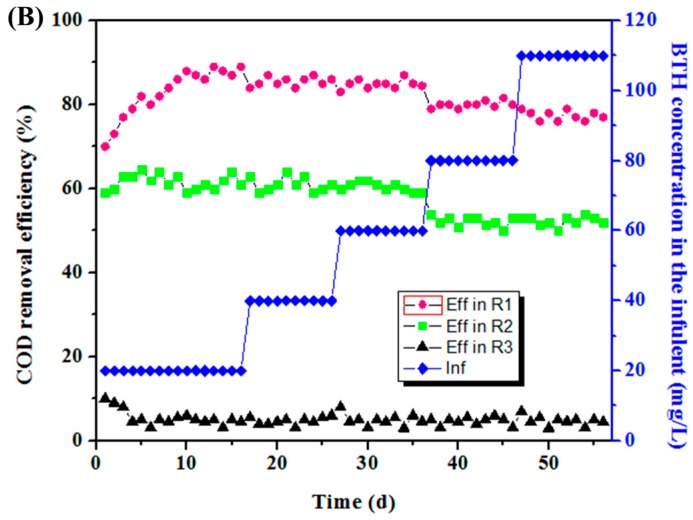
(**A**) Benzothiazole (BTH) degradation rate and (**B**) COD (Chemical Oxygen Demand) removal efficiency in the three reactors.

As shown in Figure 2, the influent BTH concentration increased from 20 mg/L to 110 mg/L, the BTH degradation rate fluctuated slightly in R1, while it decreased rapidly in R2. The BTH degradation in R3 was always at a low level. During the first few days of the experiment in R1 and R2, both the rate of COD removal and BTH degradation increased slowly, suggesting that the biodegradation ability of microbes could be strengthened after domestication, indicating that the microorganisms corresponding to these bands gradually adapted to the BTH wastewater. When the influent BTH concentration increased to 80 mg/L, both the efficiency of COD removal and BTH degradation experienced a significant reduction, indicating a relatively high concentration of BTH has an inhibiting effect of microorganism both in the MEC and anaerobic reactor. As a control, there was appreciable removal of BTH and COD in R3, which was likely due to the adsorption, volatilization, and precipitation of graphite particles.

As influent BTH increased gradually to 110 mg/L, the mean BTH degradation rate was 80.4% in R1, while it dropped to 45.3% in R2 and 4.9% in R3; similar results were observed at other influent concentrations. These results implied that at higher concentrations, BTH breakthrough, inactivation of the sludge, and inhibition of fermentation occurred, and at lesser concentration levels, BTH inhibited luminescence processes in *Vibrio fischeri*; the effects were concentration-independent and more pronounced than the growth inhibitory effects on *Vibrio fischeri* [35]. Similar results were obtained when BTH influent concentrations exceeded the maximum permissible levels [1].

As shown in Figure 2B, during the whole experiment of 56 days, there was a significant difference in the COD removal between R1 and R2. When the BTH concentration increased to 110 mg/L, the COD removal efficiency in R1 decreased gradually to 81.1% on day 56, which was obviously higher than the sum of R2 (40.4%) and R3 (3.3%). These results suggested that there was a coupling effect between the electrochemical process and biological degradation, which was mainly responsible for the enhanced performance of the reactor with respect to BTH degradation and COD removal. The improved COD removal might be ascribed to the effect of anode oxidation. It is well known that microbes on the anode biofilm can oxidize small molecular VFAs, which could relieve the accumulation of VFAs and drive the COD removal [27]. Additionally, the degradation of BTH in R1 also contributed to the improved COD removal, which can be evaluated by the difference of the COD removal rate between reactor R2 and R3.

Among the three reactors, R1 showed the highest COD removal on day 56. As compared with the reactor R2, with the extra power consumption of 0.0175 W (voltage U = 0.7 V, current I = 25 mA), reactor R1 presented a higher COD removal of 25%. The improved COD removal might be ascribed to the effect of anode oxidation. 

Proper electric stimulation can accelerate the growth of microbes via promoting microbial metabolism [36]. This speculation was confirmed by the following real-time PCR analysis.

### 3.2. Effects of Supplied Voltages on the Enrichment of Bacteria and Sludge Characteristics

These results suggested that the electric field could significantly improve the performance of this hybrid reactor. Together with the COD removal and BTH degradation of R1, it indicated that the electricity alone was not the main reason for the improved performances in the MEC reactor. The coupling between electricity and microorganisms might play a key role in the enhanced treatment.

The applied voltage could promote sludge to form a stable structure so as to increase the EPS content [37]. As shown in Table 1, the level of EPS, including proteins and polysaccharides, extracted from the sludge of reactor R1 was significantly higher than the levels in reactor R2 and the seed, and its content on the electrodes was higher than that in the sludge (anode: 49.15 mg/g VSS; cathode: 44.81 mg/g VSS). The microbial EPS mainly consisted of proteins and polysaccharides, which immobilized the microbial cells on the electrode surface by causing them to anchor and form a high cell density and by allowing direct interspecies electron transfer to facilitate cell-to-cell contact [38,39]. High EPS content in the sludge of R1 was expected to enrich more types of microorganisms in the reactor [40].

**Figure 3:**
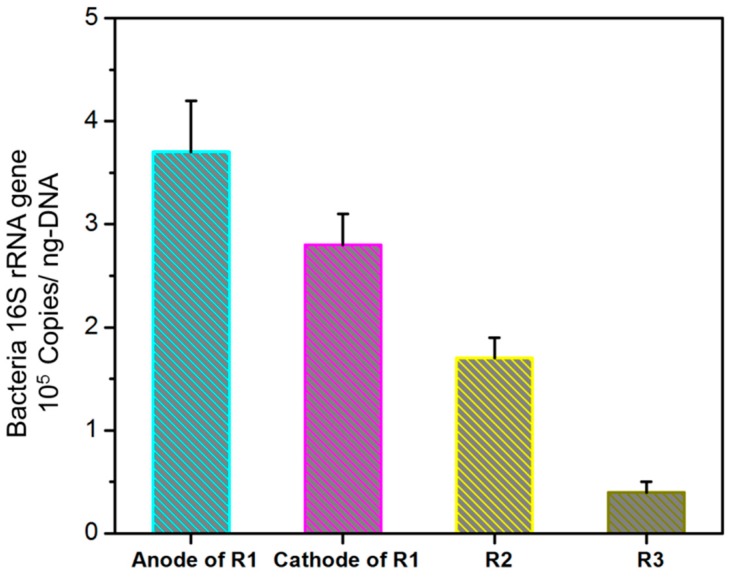
Real-time PCR quantification of total bacteria in the sludge samples of anode and cathode biofilm of R1 (MEC (Microbial Electrolysis Cell) reactor with external power and biomass), R2 (the same MEC reactor as R1, but without external power), and R3 (the same MEC reactor as R1, but without biological sludge).

The higher rate of COD removal and degradation of BTH by R1 was in line with its high EPS content, indicating that the presence of an electric field helped to accelerate the formation of EPS. As shown in Figure 3, analysis of real-time PCR quantification of bacteria showed that the numbers of copies of the Bacteria 16S rRNA gene were 3.71 ± 0.52 × 10^5^ and 2.78 ± 0.52 × 10^5^ copies/ng-DNA in the anode and cathode biofilm of R1 reactor, respectively, which was significantly higher than that in the bottom sludge of R2. In addition, the abundance of bacteria in the anode biofilm also was 1.33-folds higher than that in the anode biofilm of R1, even though both samples were in the same reactor. These results indicated that the addition of an electric field might be favorable for the enrichment of bacteria. It is believed that proper electric stimulation can promote microbial metabolism [36]. Further, high levels of EPS were favorable for cell aggregation which was also a major reason for the high abundance of bacteria in the R1 and electrode biofilm.

### 3.3. Effects of BTH Concentration on Current Value and VFA Production

**Figure 4:**
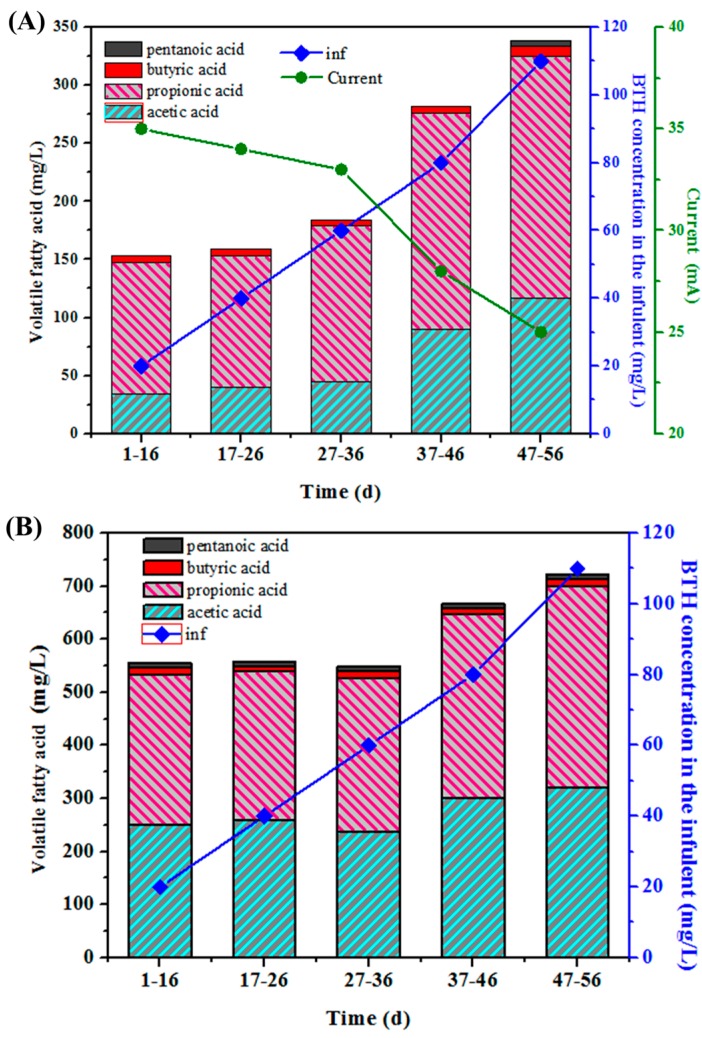
(**A**) Effluent volatile fatty acid concentrations and current value in R1 and (**B**) Effluent volatile fatty acid concentrations in R2 under different BTH concentration in the influent.

VFA is widely considered to be a process indicator during anaerobic processes, because it is the main pre-methanogenic intermediate [37]. The increase of organic loading easily resulted in the accumulation of VFA which can indicate a process imbalance). As shown in Figure 4, during the whole experiment period, the mean VFA concentrations in R1 ranged from 150 to 340 mg/L, which was significantly lower than that of 525 to 720 mg/L in R2. This is because microbial oxidation of organic material at the anode (e.g., acetate oxidation) liberates electrons (e^−^) and protons (H^+^), which undergo chemical reduction at the cathode to form H_2_. Under standard temperature and pressure conditions (STP: 273 K, 1 atm, adjusted to pH 7.0) [41], the reduction potential at the anode is higher than at the cathode, and thus a small voltage must be supplied to drive this non-spontaneous reaction. However, with the exception of glucose, the reduction potential of the anodic half-reaction is usually much higher [27]. In practice, this potential difference is even greater due to resistance in the system and polarization at the electrodes. Consequently, in order to drive electrons from the anode to the cathode and overcome this thermodynamic limit for the mineralization of VFAs, energy from an external power source must be supplied to the cell. The relative low level of VFA in R1 was attributed to the enhanced microbial reduction due to the current. The introduction of the anode in R1 further accelerated the mineralization of VFAs, which led to the lowest level of VFAs in the effluent.

According to the stoichiometry of the following reactions (Wrana, Sparling et al., 2010):

C_6_H_12_O_6_ + 2H_2_O→2CH_3_COOH + 4H_2_ + 2CO_2_ (1)

CH_3_COOH + 2H_2_O→2CO_2_ + 8H^+^ + 8e^−^ (2)

converting 1 mol of glucose would produce 2 mol of acetic acid along with the production of hydrogen and carbon dioxide, the direct contribution of the supplied current (about 25 mA to 35 mA) in R1 to the COD removal efficiency was lower than 2% (about 0.86% to 1.2%), which was negligible. Nevertheless, the electricity enhanced process for improving the performance of anaerobic fermentation might be slow due to the low metabolism of anaerobic bacteria, especially methanogens. Even though there were no obvious and immediate effects for COD removal and/or less residual VFAs, it might function well after a period of acclimation under the coupling effect of electricity and microorganism. When the voltage was cut off in R1 at the end of the experiment, effluent VFAs in the R1 reactor increased gradually to 640 mg/L (data not shown), which further confirmed the positive effect of the electric field for the decomposition of VFAs and the removal of COD. This result was in agreement with previous reports [32,37,42].

On the other hand, the TVFA in the three reactors tended to accumulate gradually with the increase of influent BTH concentration from 20 to 110 mg/L. During the initial 36 days, the mean VFA concentrations in R1 ranged from 150 to 180.6 mg/L at an influent BTH concentration of 20 to 60 mg/L. As the influent BTH concentration increased from 80 to 110 mg/L, the VFA concentration in R1 increased to 342.1 mg/L on day 56, and R2 showed the same trend.

As the influent BTH increased gradually from 20 to 110 mg/L, the current decreased from 35 to 25 mA. The increased influent BTH can lead to more VFAs accumulation via depressing the anaerobic fermentation process and direct anode oxidation process that can be further used by exoelectrogens on anode biofilm. More production of VFAs could provide more substance for the growth of exoelectrogens, which was likely to explain the decrease in current.

### 3.4. Antibacterial Activity Elimination Due to BTH Concentration

**Figure 5:**
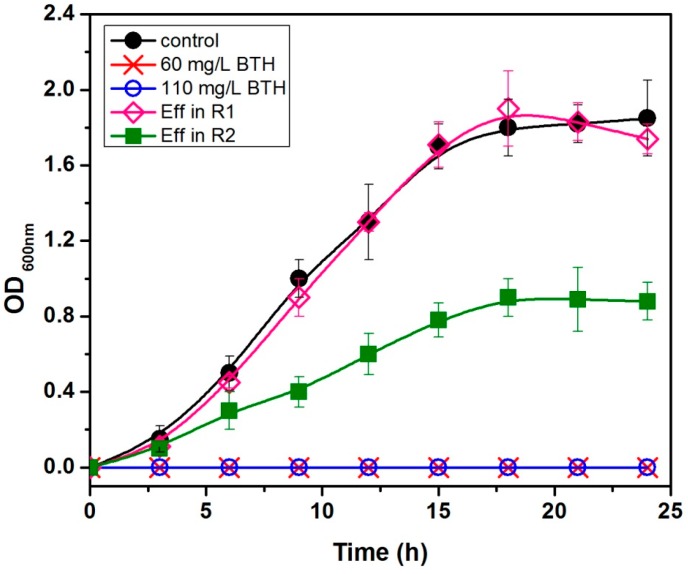
Effect of BTH (0, 60, and 110 mg/L) and the intermediate metabolite on the growth of *S. oneidensis* MR-1.

The antibacterial activity assay was performed using selected bacterial strains (*S. oneidensis* strain MR-1). The growth of the strain was completely inhibited in the presence of BTH at 0, 60, and 110 mg/L, and the effluent of R1 and R2 with the influent BTH of 60 mg/L (Figure 5). This observation suggests that the end product of BTH in R1 has much less antibacterial activity than R2.

As the end product of BTH in the R1 reactor is less toxic to microorganisms and relatively easier to be biodegraded, thus, the reduction of BTH using a microbial electrochemical method would have ecological significance in the elimination of the selection pressure of antibiotic for the generation of antibiotic resistant bacteria/genes in the environments. Similar observations by other researchers reducing the chlorinated nitroaromatic antibiotic chloramphenicol by biocathode showed the same results [43,44].

On the other hand, BTH with different concentrations, and the intermediates of BTH reduction in R2, are toxic to microorganisms, and can retain their antibacterial activity in other kinds of aromatic antibiotic derivatives. Data in the literature for luminescence inhibition of BTH (EC50, 5 mg/L) are slightly lower than those determined here [35]. This is not astonishing, since the luminescence test monitors physiological activity rather than mortality. As mentioned above, the microbial electrochemical process is likely superior to microbial technology in the elimination of antibacterial activity from BTH-containing wastewaters.

## 4. Conclusions

(1) An up-flow internal circulation microbial electrolysis reactor (UICMER) was shown to be an effective method for BTH degradation. Benzothiazole and its intermediates can be degraded completely to simple organic acids by MEC treatment.
(2) In UICMER, the electric field significantly improved the treatment performance for high concentration BTH wastewater in both COD removal and BTH degradation with intermediate biodegradability, and led to more EPS production, which was favorable for the enrichment of bacteria in the anode and cathode biofilm.
(3) The right amount of VFA production could provide more substance for the growth of exoelectrogens; the presence of an electric field improves the degradation of BTH in UICMER.
(4) The reduction of BTH using a microbial electrochemical method would have ecological significance in the elimination of the selection pressure of antibiotic for the generation of antibiotic resistant bacteria/genes in the environments.

As mentioned above, the microbial electrochemical process is likely superior to microbial technology in the elimination of antibacterial activity from BTH-containing wastewaters.

## Figures and Tables

**Table 1:** Total extracellular polymeric substances (EPS) in R1, R2, electrode surfaces and seed sludge on day 56.

Sample	EPS		
	Polypeptides	Polysaccharides	Sum
	(mg Protein/g VSS)	(mg Glucose/g VSS)	(mg EPS/g VSS)
Seed sludge	11.23 ± 0.69	7.8 ± 0.41	19.03 ± 1.1
Sludge of R1	23.43 ± 0.87	15.58 ± 0.57	39.01 ± 1.44
Sludge of R2	13.34 ± 0.78	8.89 ± 0.37	22.23 ± 1.15
Anode surface	29.9 ± 1.02	19.25 ± 0.67	49.15 ± 1.69
Cathode surface	27.3 ± 0.89	17.51 ± 0.48	44.81 ± 1.37
